# Application of a Flow-Based Hollow-Fiber Co-Culture System to Study Cellular Influences under Hyperglycemic Conditions

**DOI:** 10.1038/s41598-019-40555-0

**Published:** 2019-03-07

**Authors:** Abdul Shukkur Ebrahim, Thomas W. Carion, Eliisa Strand, Laura A. Young, Haoshen Shi, Elizabeth A. Berger

**Affiliations:** Department of Ophthalmology, Visual & Anatomical Sciences Wayne State University School of Medicine Detroit, Detroit, MI 48201 USA

## Abstract

Elucidation of the basic mechanisms underlying human disease pathogenesis depends on the findings afforded to us through *in vivo* and *in vitro* approaches. While there are inherent limitations in any model system, 2D *in vitro* culture systems tend to be particularly restricted due to their static nature. Here, we adapted a flow-based hollow-fiber cartridge system to better understand the cellular influences of human retinal microvascular endothelial cells and mouse-derived neutrophils under high glucose conditions similar to those observed in diabetes. Analyses by western blot and flow cytometry indicate that pro-inflammatory molecules known to be associated with the pathogenesis of diabetic retinopathy were significantly elevated following high glucose exposure, including VEGF, ICAM-1, and ROS. Changes in mitochondrial potential were also observed. Further, we demonstrate that this innovative system allows for cross-species co-culture as well as long-term culturing conditions. This *in vitro* modeling system not only mimics the retinal microvasculature, it also allows for the examination of cellular interactions and mechanisms that contribute to diabetic retinopathy, a visually debilitating complication of diabetes.

## Introduction

Despite increased understanding of potential mechanisms of disease pathogenesis, diabetic retinopathy (DR) remains the leading cause of blindness. It is estimated that approximately 1/3 of the 285 million diabetics worldwide present with signs of retinopathy^1^. To date, only anti-VEGF therapy has emerged as an effective approach, yet it is limited by its use via intravitreal injection and effectiveness for only a subset of advanced patients with diabetic macular edema and/or proliferative retinopathy. Additional therapies that work with VEGF or target adjunctive pathways are needed – notably for early stages of disease. In this regard, the way we look at DR has broadened with the role of inflammation becoming more widely recognized in the last decade as a key contributor. Not only have studies demonstrated increased leukostasis in the diabetic retina^2,3^; but, physiologic and molecular events that are consistent with inflammation and the innate immune pathway have been observed in retinas of both diabetic patients and animals^4^. In fact, a number of inflammatory mediators and pathways, including NF-κB, TNF-α, IL-1β, and COX-2 have been shown to contribute to the development of clinically recognized lesions associated with DR^3,5–8^.

Microvascular pathology is well associated with the development and progression of DR. Early stages of pathology include (but are not limited to): acellular capillary formation, cell death (both retinal endothelial cells [RECs] and associated pericytes), subclinical inflammation and leukostasis^9^. Resident RECs are among the first to interact with infiltrating inflammatory cells. It has also been shown that RECs can produce and respond to several inflammatory mediators^10–12^. The adherence of inflammatory cells such as neutrophils (PMNs) to RECs leads to increased permeability of retinal vasculature, further augmenting the production of inflammatory mediators and infiltration of additional inflammatory cells in the diabetic retina. Loss of RECs occurs subsequent to neuronal changes in non-proliferative retinopathy; yet, it is this vascular damage (perhaps largely inflammation induced) that precedes the development of vision-threatening proliferative DR.

Given the close interaction between RECs and infiltrating inflammatory cells in the retinal vasculature during the progression of DR, it is imperative to generate *in vitro* conditions that closely mimic what is observed *in vivo* in order to appropriately examine this interrelationship. Currently, *in vitro* analysis is largely limited to traditional 2D static culture conditions, which poorly mimic the dynamic environment within the human body. Further, examination of cellular infiltrates such as PMNs are especially limited as these cells tend to undergo apoptosis within 24–48 h unless differentiation or growth factors are present. Taken together, the reliability and significance of data obtained from such approaches are narrow and inherently limited. As such, the current study uses the flow-based hollow-fiber modeling system, which is modeled after the mammalian circulatory system and mimics retinal vasculature conditions *in vivo* to better understand how this cellular interaction and high glucose conditions contribute to DR pathogenesis. Advantages of the current system include reduced apoptosis and improved cell function despite extended, long-term culture conditions. Although hollow fiber bioreactors are not a new technique per se, this is the first study to establish a co-culture system between HREC and mouse-derived PMN under such a physiologically dynamic environment.

## Results

### VEGF levels in HREC are increased under HG conditions

**Figure 1:**
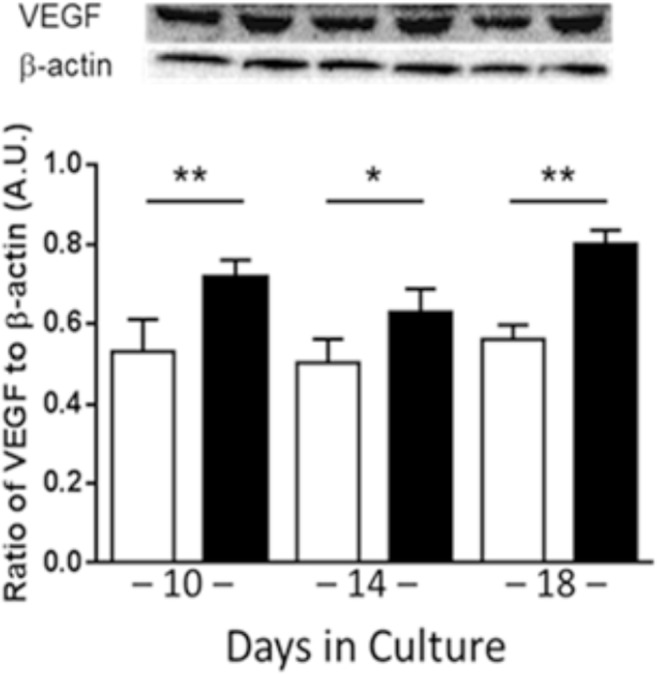
VEGF levels as detected by Western blot in HRECs after 10, 14 and 18 days cultured alone in normal glucose (NG, white) or high glucose (HG, black) using the flow-based hollow-fiber system. Blots are shown as cropped images. *N* = 3/group; results are expressed as mean ± SD. *P < 0.05, **P < 0.01.

Using the flow-based hollow-fiber system, we first cultured human retinal endothelial cells (HRECs) alone to establish that these cells are responding to normal and high glucose conditions similarly to that which is observed under static culture conditions and in *in vivo* murine models of streptozotocin (STZ)-induced DR. As shown in Fig. 1, VEGF levels, as detected from culture supernatants by Western blot, were significantly increased with high glucose exposure at 10, 14 and 18 days compared to normal glucose controls. Cell viability was maintained over time at >85%, as detected daily by trypan blue staining (data not shown).

### HREC response to HG conditions after co-culture with mouse-derived PMNs

**Figure 2:**
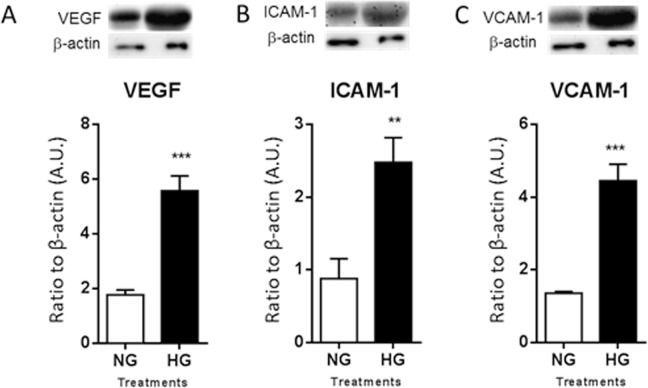
Protein levels of human VEGF (**A**), ICAM-1 (**B**), and VCAM-1 (**C**) as detected by Western blot in cellular lysates. HRECs were co-cultured with PMNs for 28 days under normal glucose (NG) or high glucose (HG) conditions. One representative cropped image is provided for each Western blot. *N* = 5/group; results are expressed as mean ± SD. **P < 0.01, ***P < 0.001.

After two weeks of acclimation, murine PMNs were added to the culture system under normal and high glucose conditions. Following 28 days of co-culture, HRECs response was analyzed by Western blot for select pro-inflammatory mediators known to be elevated during DR (Fig. 2). Levels of VEGFB (***A***), ICAM-1 (***B***) and VCAM-1 (***C***) were significantly upregulated in cellular lysates after exposure to HG versus NG.

### PMN response to HG conditions after co-culture with HRECs

**Figure 3:**
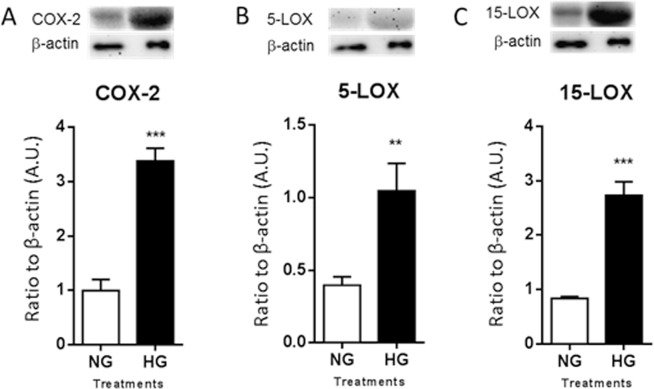
Protein levels of COX-2 (**A**), 5-LOX (**B**), and 12/15-LOX (**C**) in PMNs as detected by Western blot. PMNs were co-cultured with HRECs for 28 days under normal or high glucose conditions. One representative cropped image is provided for each Western blot. *N* = 5/group; results are expressed as mean ± SD. *P < 0.05, **P < 0.01, ***P < 0.001, ****P < 0.0001.

Cellular lysates were examined after 28 days of co-culture in normal and high glucose for expression of mouse COX-2 (***A***), 5-LOX (***B***), and 12/15-LOX (***C***), as shown in Fig. 3. All three molecules were significantly elevated under HG compared to NG conditions. COX-2 is an enzyme that is rapidly upregulated during inflammation. In addition, 5-LOX is a marker of inflammatory leukocytes. Elevated levels of both enzymes are accordant with an amplified inflammatory response. While 12/15-LOX is an enzymatic marker of proresolving pathway activity.

In addition, PMN cell viability was determined daily by trypan blue staining. Viability was markedly increased at 3 days of culture using the flow-based hollow-fiber system (>70%) compared to the traditional 2D culture dish (<10%). In fact, PMNs maintained >70% viability through 28 days when the system was terminated for further analysis.

### Elevated oxidative stress levels in response to HG conditions

**Figure 4:**
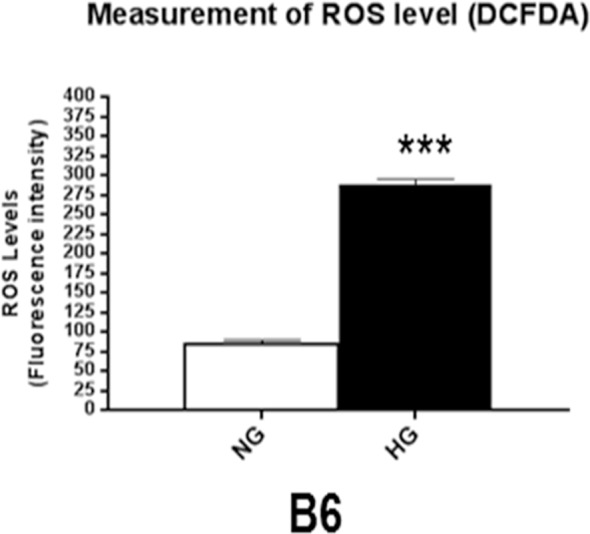
ROS levels were determined by fluorescent values measured from cellular lysates after 28 days of co-culture under normal or high glucose conditions. Fluorescent values were acquired by subtraction of negative control values from 2′, 7′-dichlorofluorescin diacetate values. *N* = 6/group; results are expressed as mean ± SD. ***P < 0.001.

Increased oxidative stress is known to play a pathogenic role in diabetes-induced complications, including DR^13,14^. In fact, it has been indicated that oxidative stress contributes to both the development of retinopathy and its persistence following restoration of glycemic control^13^. DCFH-DA detects most ROS, including one-electron-oxidizing species, heme proteins, oxidation-reduction-active metals, and hydrogen peroxide. As indicated in Fig. 4, ROS levels were significantly elevated after HG exposure as detected from cell lysates obtained after 28 days of co-culture.

**Figure 5:**
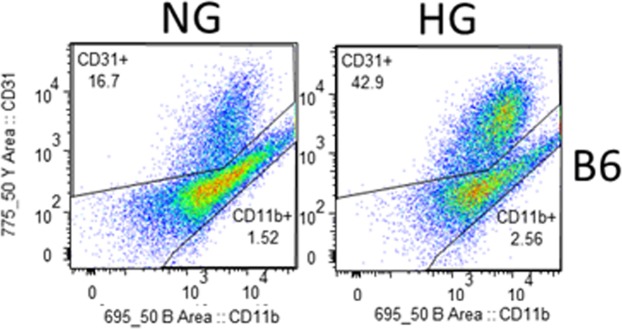
Representative flow cytometry plots denoting the gating strategies used to identify HRECs and PMNs after 28 days of co-culture under normal glucose and high glucose conditions. CellROX Green staining was used to detect ROS levels, while TMRM staining was used to detect membrane potential.

In addition, flow analysis was used to assess ROS levels and membrane potential under NG and HG conditions after 28 days of co-culture. As indicated in the gating strategy presented in Fig. 5, CD31^+^ cells were identified as HRECs and CD11b^+^ cells as PMNs. Mean fluorescent intensity values (Table 1) indicated that ROS levels were increased by 32.9% in HRECs and 51.6% in PMNs when exposed to HG compared to NG. Hyperglycemia-induced mitochondrial dysfunction and resultant loss of membrane potential further contributes to sustained, chronic overproduction of ROS. Correspondingly, membrane potential was reduced by 25.4% in HREC and 6.9% in PMNs after HG exposure compared to control conditions.

**Table 1:** Fluorescent intensity values as obtained by flow cytometry.

	CD31+		CD11b+	
	ROS	Membrane potential	ROS	Membrane potential
NG	3797	3326	1794	138
HG	5046	2479	2721	1284

## Discussion

Understanding the cellular interactions under hyperglycemic conditions will provide insight into the retino-immunopathogenic events associated with this visually debilitating disease. Cell culture models are an essential tool, used in combination with *in vivo* animal models, to elucidate the basic science underlying human disease pathogenesis. However, *in vitro* studies are inherently limited regarding aspects such as capturing interactions between different cell types and assessing the effects of long term exposure. To this end, we sought to examine an *in vitro* co-culture system that utilizes flow-based hollow fiber cartridges to mimic *in vivo* retinal vasculature conditions; thus, allowing us to examine the cellular interactions between REC, a residential retinal cell, with an infiltrating innate immune cell, PMN, under hyperglycemic conditions. The flow-based hollow-fiber cartridge system allows for the culturing and co-culturing of cells in an *in vitro* environment that better reflects the physiological conditions of a mammalian system. Further, as cells are bound to a porous support, they do not require passaging and can be maintained for extended time courses without significant apoptosis observed under static culturing conditions.

Vascular pathology, including microvascular leakage and neovascularization, has been considered a major feature of the pathogenesis related to diabetic retinopathy. Retinal microvascular damage is largely induced by inflammatory cells, particularly leukocyte-mediated retinal non-perfusion, cell junction disruption and apoptosis of pericytes and retinal endothelial cells^4^. In fact, we recently showed that hyperglycemic conditions alone are somewhat limited in inducing an inflammatory response in HRECs^15^. But that these cells were (differentially) influenced by the presence of cytokines, supporting the idea that innate immune cells (a major source of inflammatory mediators) potentiate the effects of diabetic conditions. The flow-based hollow-fiber cartridge system is extremely beneficial for studying cellular diseases as it better reflects the *in vivo* environment compared to traditional static culture conditions, as demonstrated herein for HRECs in response to high glucose conditions and in the presence of other cells, namely PMNs. In fact, the lifespan of PMNs is a major limiting factor in studying their function *in vitro*^16,17^ – an aspect that the current co-culturing system markedly improves.

Studies have implicated mitochondrial dysfunction in the disruption of whole-body metabolic homeostasis^18^. Increased oxidative stress has been shown to damage mitochondrial function, resulting in retinal metabolic abnormalities^19,20^. It has been established that oxidative stress contributes to hyperglycemia-induced retinal vascular cell injury^21^. Our results are in agreement that RECs contribute to this dysfunction; and further, that PMNs may either be protective or perhaps less metabolically vulnerable to high glucose-induced damage. These findings suggest the extent to which this co-culturing system can be manipulated to understand the scope of cellular involvement in the metabolic dysfunction associated with development of diabetic retinopathy.

Furthermore, the advantages of this system over static 2D culture conditions go beyond our current findings. Not only are RECs exposed to chronic shear forces during culture, but they are able to lay flat and form traditional monolayers with tight junctions, all of which creates a more physiologically relevant environment under which to study these cells. The retinal microvasculature is a major site of pathology related to diabetic retinopathy^19^. RECs maintain the blood retinal barrier with support of pericytes at a 1:1 ratio^14^. Chronic exposure to high glucose conditions has been shown to shift this ratio. In addition to the current body of work regarding retinal pericytes^22–25^, future work using the 3D cell culture cartridge system will contribute to the comprehensive investigation into the role of these support cells – with the potential to shed light on diabetes-induced retinal pathology, such as vascular leakage and neovascularization. We anticipate that use of this co-culture system will allow for expanded investigation into retinal glial cells and other infiltrating inflammatory cells, as well.

Understanding the complex mechanisms of diabetic retinopathy is imperative in the development of potential treatments to delay or reverse this disease. The innovative flow-based hollow-fiber co-culture system advances this knowledge and provides insight into the *in vivo* environment associated with diabetes in the retina.

## Methods

### Human retinal microvascular endothelial cell culture

Primary HRECs (Cell Systems Corporation; Kirkland, WA) were grown in HREC medium containing microvascular growth supplements (MVGS; Invitrogen, Carlsbad, CA), 10 mg/mL gentamicin, and 0.25 mg/mL amphotericin B. Cells were used within six passages. For cell seeding of the culture system, two confluent T75 flasks were prepared for each cartridge. HRECs were rinsed 2× with PBS, trypsinized and resuspended at a concentration of 1.5 × 10^7^ cells in 1–2 mL media prior to loading into the artificial capillary technology cartridge system (FiberCell Systems, Frederick, MD) described below. Cells were derived from the same passage for each experiment, which consisted of two cartridge systems – one normal glucose and one high glucose.

### PMN isolation

Peritoneal PMNs were isolated from female C57BL/6 (B6) mice as previously described^26,27^. All experimental protocols were approved by the Institutional Animal Care and Use Committee at Wayne State University (Protocol#16-05-090) and were carried out in accordance with the Association for Research in Vision and Ophthalmology’s statement on the Use of Animals in Ophthalmic and Vision Research. Briefly, mice received a 1.0 mL intraperitoneal injection of a 9% casein solution at 27 and 3 h prior to cell harvest. Cells were lavaged from the peritoneal cavity 3 h following the second injection, then collected in harvest solution (0.02% EDTA in 1× PBS), washed, and isolated using a 90% Percoll gradient. Viable cells (>95%) were quantitated and resuspended in either high-glucose (25 mM) or normal-glucose (5 mM) medium (DMEM medium supplemented with glucose).

### Flow-based hollow fiber co-culture cartridge system

**Figure 6:**
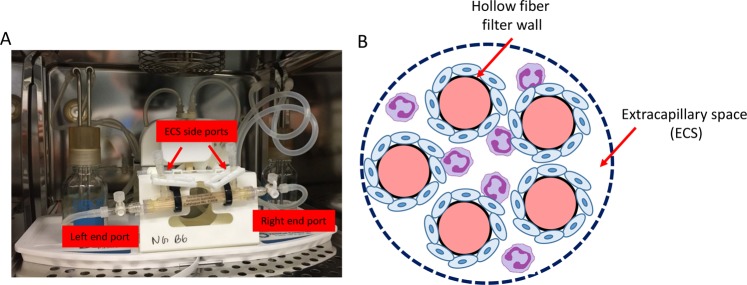
Photograph of co-culture system (**A**), which is further depicted schematically (**B**). The enlarged cross section illustrates only five of 20 total fibers. HREC (blue) are seeded into the ECS where they form a monolayer over the fibers, followed by the addition of PMN (purple). Co-culture conditions include both normal glucose and high glucose to investigate the cellular influences and interactions between the two cell types under physiological shear forces similar to that observed in the retinal microvasculature.

The commercially available flow-based hollow fiber co-culture system was purchased from Fiber Cell Systems (Fig. 6). The small cartridges (Cat # C2025) are made of PVDF with an approximate pore size of 0.1 μm and 80 cm^2^ outer surface area within an extracellular space (ECS) volume of 3.9 mL for the highest exchange rates but without protein retention. Prior to cell seeding, the PVDF fibers of the FiberCell cartridge system were activated according to the manufacturer’s recommendation. After flushing with 70% ethanol (sterile filtered) and rinsing with sterile water, the fibers were then activated (60 min) by coating with Attachment Factor^TM^ (Cell Systems Corporation; Kirkland, WA), which promotes cell attachment and encourages correct polarity and cytoskeletal organization. HRECs media was circulated to initiate pre-culture for 24 hours. Following pre-culture, freshly harvested HRECs (~1.5 × 10^7^ cells) were resuspended in a total of 5 mL of HREC medium and loaded into the lumen of the capillary fibers of each cartridge system using a syringe attached to the left end port. Cells were slowly transferred (3–5 passes) between the left and right end ports with both ECS side ports closed. The cartridge was maintained at 37 °C, 5% CO_2_ in a humidified incubator for 60 min without flow, allowing the HRECs to attach to the fibers. The cartridge was rotated 180° after 30 min.

The endothelial cells were next adapted to shear stress levels by initiating flow (10–30 pulses/min) to the luminal side of the capillary fibers for 12 hours, then gradually increased. The media was then changed to HREC media supplemented with either normal glucose (5 mM; NG) or high glucose (25 mM; HG). The initial media was retained to determine the number and viability of the cells that did not adhere to the cartridge fibers. Before initiating co-culture conditions, the system was maintained for 14–18 days under a steady laminar flow to generate ~20 dyne/cm^2^ - similar to retinal microcirculation observed in humans^28^. Mouse-derived PMN were then introduced directly to the cartridge system by adding cells (5 mL diluted to 1 × 10^6^ cell/mL) to the ECS of the fiber cartridges by flushing between the central side ports with the two end ports closed (Fig. 6). The system was maintained at 37 °C, 5% CO_2_ in a humidified incubator with media added to the reservoir bottle according to the manufacturer’s guidelines to maintain a consistent volume of 50 mL. Samples (3 mL) were obtained from the ECS and lumen, and were used to determine cell viability by trypan blue staining.

### Cell harvesting

After 28 days of co-culture, all media was collected from both the ECS and within the fibers of the cartridge system. Cells were then harvested using trypsin (0.25%, 10 mL) incubated for 5 min, followed by flushing between the end ports and side ports using syringes to collect the cells from the ECS and lumen of the capillary fibers. Media and cells were combined, centrifuged and further processed for protein analyses or flow cytometry, as described below.

### Western blot analysis

Cellular lysates were prepared for protein isolation by resuspending in lysis buffer containing protease and phosphatase inhibitors. Cellular extracts were then prepared by sonication, and total protein concentration was determined. Equal amounts of total protein (40 μg) were separated on 4–20% tris-glycine gels (Invitrogen) and transferred to nitrocellulose membranes. After blocking membranes in TBST (10 mM Tris-HCl buffer, pH 8.0, 150 mM NaCl, 0.1% Tween 20) and 5% (w/v) BSA at room temperature for 60 minutes, membranes were incubated overnight at 4 °C with antigen-specific primary antibodies. The primary antibodies were used as follows: anti-VEGF (1:1000; Abcam, Cambridge, United Kingdom); anti-ICAM-1 (1:1000) (Cell Signaling Technology, Danvers, MA); anti-VCAM-1 (1:1000; Cell Signaling Technology); anti-COX-2 (1:200; Santa Cruz, Santa Cruz, CA); anti-5-LOX (1:500; Abcam); anti-15-LOX (1:1000; Abcam) and anti-β-actin (1:1000; Santa Cruz). Blots were then incubated with species-specific HRP-conjugated secondary antibodies for 2 hours at room temperature. Proteins were visualized by incubation with a chemiluminescence substrate kit (Thermo Fisher Scientific, Waltham, MA). Western blot images were collected (Azure Biosystem C500, Dublin, CA) and target protein expression was quantified (Image Studio Lite software) after normalizing to β-actin. One representative blot is shown for each molecule.

### Measurement of ROS levels in the cellular lysates

Cellular lysates (10 μg) were incubated in reaction buffer [130 mM KCl, 5 mM MgCl_2_, 20 mM NaH2PO4, 20 mM Tris-HCl, pH 7.4, 30 mM D-glucose, 7.5 µM 2′,7′- dichlorofluorescin diacetate (DCFH-DA) (Thermo Fisher Scientific)] for 1 hour at 37 °C^29,30^. ROS oxidizes DCFH-DA, resulting in the formation of dichlorofluorescein (DCF), a detectable fluorescent product^31^. Negative controls were incubated in reaction buffer without DCFH-DA. Fluorescence levels were measured by SpectraMax M3 Multi-Mode reader (Molecular Devices, Sunnyvale, CA). The excitation and emission wavelengths used were 485 and 527 nm, respectively^29^. The final fluorescent values were acquired by calculating DCF fluorescent values minus negative control values.

### Flow cytometry analysis

Two color flow cytometry analysis was conducted on co-culture derived samples (both HREC and PMN cells) to identify CD31^+^ and CD11b^+^ cells. Cells were collected from the ECS and lumen of the capillary fibers and centrifuged for 5 minutes at 300 g at room temperature. Cell pellets were washed using phosphate-buffered saline (PBS) and then suspended in cold PBS. After performing cell counts and viability using trypan blue, cells were incubated with antibody CD31-PE and CD11b-APC-Cy™7 (BD Biosciences, San Jose, CA) using the manufacturer’s recommended volumes and incubation conditions. Cells were washed briefly in PBS and the pellets were resuspended in 1 mL fresh cold PBS. The fluorescence intensity of CD31 and CD11b antigens of 10,000 cells were analyzed after gating on intact cells using forward and light-scatter patterns by flow cytometry (SH800 cell sorter, Sony Biotechnology).

### Reactive oxygen species (ROS) and mitochondrial potential (Δψ_m_) analysis

Production of ROS was assessed in living cells using the fluorogenic probe CellROX^®^ Green reagent (Molecular Probe), a cell-permeable dye that is non-fluorescent when in a reduced state, while exhibiting strong fluorescent signal upon oxidation, with excitation/emission maximum of ∼485/520 nm. Briefly, cells (2 × 10^5^/mL) were resuspended in PBS, CellROX green (5 µM) and TMRM (tetramethyl rhodamine methyl ester 10 nM, mitochondrial potential Δψ_m_ marker) sensitive probe were added and then cells were incubated at 37 °C in a water bath for 30 minutes. At the end of the incubation, cells were acquired on a SY3200 cell sorter (Sony Biotechnology). In general, a minimum of 10,000 events/cells were acquired.

### Statistical analysis

All assays were carried out from at least three independent experiments and the data are presented as mean ± SD, unless otherwise noted. Data were acquired in a blinded fashion and analyzed by the Analysis of variance (ANOVA) test with Tukey’s post-hoc test and multiple comparison. *P < 0.05, **P < 0.01, ***P < 0.001, ****P < 0.0001 were considered statistically significant.

## Supplementary information

Supplementary Information

## Data Availability

All data generated during and/or analysed during the current study are included in this published article.
