# Supplementary material for: Application of a Flow-Based Hollow-Fiber Co-Culture System to Study Cellular Influences under Hyperglycemic Conditions
Source: Sci Rep. 2019 Mar 7;9:3771. doi: 10.1038/s41598-019-40555-0 (PMC6405855; doi:10.1038/s41598-019-40555-0)
Supplement: Supplementary file 1 — Supplementary Information [file 41598_2019_40555_MOESM1_ESM.pdf]

Supplementary Information

Application of a Flow-Based Hollow-Fiber Co-Culture System to Study Cellular Influences under Hyperglycemic Conditions

Abdul Shukkur Ebrahim, Thomas W. Carion, Eliisa Strand, Laura A. Young, Haoshen Shi and Elizabeth A. Berger

Figure S1: Images of full length blots of VEGF (**A**) and β-actin (**B**) in HRECs cultured in normal and high glucose using the flow-based hollow-fiber system. Lanes 1, 3, 5 = normal glucose; lanes 2, 4, 6 = high glucose. These images are shown as cropped in Figure 1.

Figure S2: Images of full length blots of VEGF (**A**), ICAM-1 (**B**), VCAM-1 (**C**), and β-actin (**D**) in HRECs cultured in normal and high glucose using the flow-based hollow-fiber system co-cultured with mouse-derived PMN. Lanes 1, 3, 5 = normal glucose; lanes 2, 4, 6 = high glucose. These images are shown as cropped in Figure 2.

Figure S3: Images of full length blots of COX-2 (**A**), 5-LOX (**B**), 12/15-LOX (**C**), and β-actin (**D**) in PMNs cultured in normal and high glucose using the flow-based hollow-fiber system co-cultured with HRECs. Lanes 1, 3, 5 = normal glucose; lanes 2, 4, 6 = high glucose. These images are shown as cropped in Figure 3.

Figure S1.

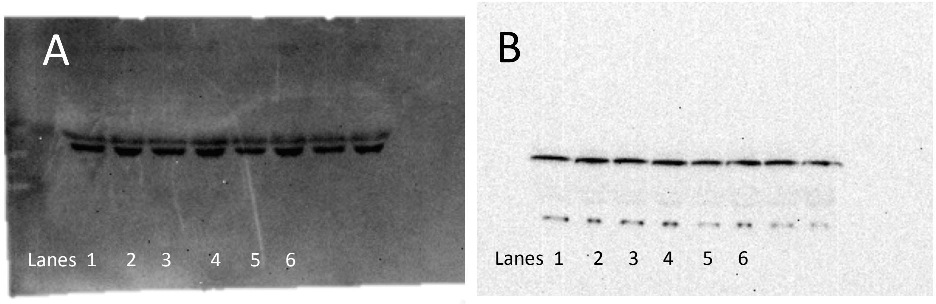

Figure S2.

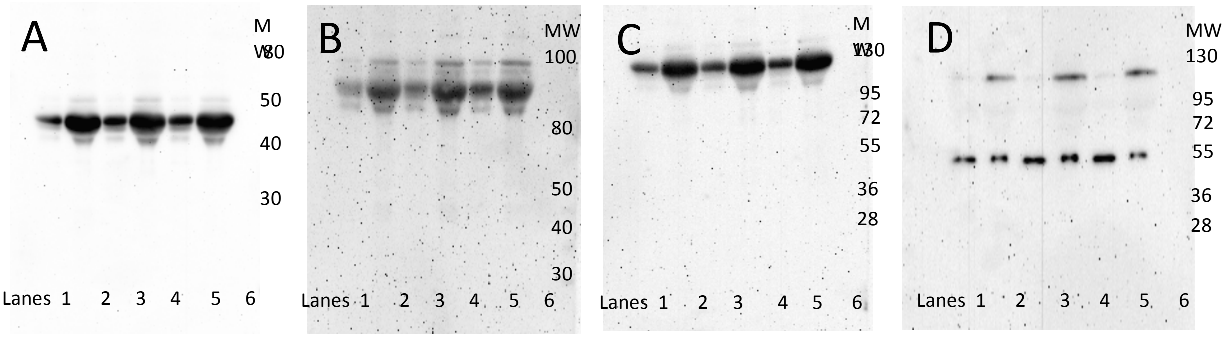
A
M
80
50
40
30
Lanes 1 2 3 4 5 6
B
MW
100
80
50
40
30
Lanes 1 2 3 4 5 6
C
130
95
72
55
36
28
Lanes 1 2 3 4 5 6
D
MW
130
95
72
55
36
28
Lanes 1 2 3 4 5 6

Figure S3.

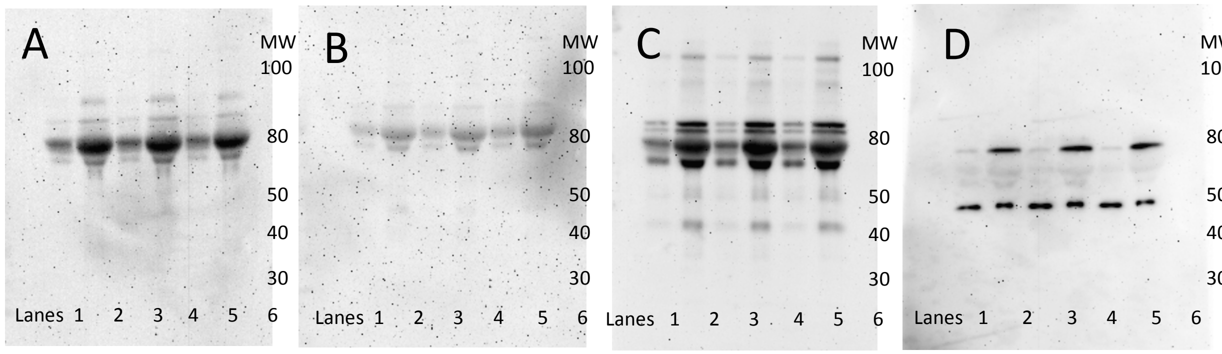
A
MW
100
80
50
40
30
Lanes 1 2 3 4 5 6
B
MW
100
80
50
40
30
Lanes 1 2 3 4 5 6
C
MW
100
80
50
40
30
Lanes 1 2 3 4 5 6
D
MW
100
80
50
40
30
Lanes 1 2 3 4 5 6
